# Association between JAK2 rs4495487 Polymorphism and Risk of Budd-Chiari Syndrome in China

**DOI:** 10.1155/2015/807865

**Published:** 2015-10-18

**Authors:** Peijin Zhang, Yanyan Zhang, Jing Zhang, Hui Wang, He Ma, Wei Wang, Xiuyin Gao, Hao Xu, Zhaojun Lu

**Affiliations:** ^1^Department of Public Health, Xuzhou Medical College, Xuzhou, Jiangsu 221004, China; ^2^Department of General Practice, Xuzhou Medical College, Xuzhou, Jiangsu 221004, China; ^3^Department of Community Health Care, Huai'an Maternal and Child Health Hospital, Huai'an, Jiangsu 223002, China; ^4^Department of Interventional Radiology, Affiliated Hospital of Xuzhou Medical College, Xuzhou, Jiangsu 221002, China

## Abstract

Myeloproliferative neoplasms (MPNs) are the leading cause of Budd-Chiari syndrome (BCS), and the C allele of JAK2 rs4495487 was reported to be an additional candidate locus that contributed to MPNs. In the present study, we examined the role of JAK2 rs4495487 in the etiology and clinical presentation of Chinese BCS patients. 300 primary BCS patients and 311 healthy controls were enrolled to evaluate the association between JAK2 rs4495487 polymorphism and risk of BCS. All subjects were detected for JAK2 rs4495487 by real-time PCR. *Results*. The JAK2 rs4495487 polymorphism was associated with JAK2 V617F-positive BCS patients compared with controls (*P* < 0.01). The CC genotype increased the risk of BCS in patients with JAK2 V617F mutation compared with individuals presenting TT genotype (OR = 13.60, 95% CI = 2.04–90.79) and non-CC genotype (OR = 12.00, 95% CI = 2.07–69.52). We also observed a significantly elevated risk of combined-type BCS associated with CC genotype in the recessive model (OR = 4.44, 95% CI = 1.31–15.12). This study provides statistical evidence that the JAK2 rs4495487 polymorphism is susceptibility factor JAK2 V617F positive BCS and combined BCS in China. Further larger studies are required to confirm these findings.

## 1. Introduction

Primary Budd-Chiari syndrome (BCS) is an uncommon condition characterized by a blocked hepatic venous outflow tract at various levels from small hepatic veins to inferior vena cava, resulting from thrombosis or its fibrous sequel [1]. In Western hemisphere, BCS is a rare disease with an annual incidence of around 1-2 per million inhabitants [2], predominantly affecting young females [3, 4]. It is closely associated with underlying thrombotic risk factors including myeloproliferative neoplasms (MPNs), factor V Leiden mutation, factor II mutation, hyperhomocysteinemia, and paroxysmal nocturnal hemoglobinuria [1, 5–9]. By contrast, China has substantially larger numbers of BCS patients [10], but the etiology is still in its infancy. These well-known risk factors are rarely observed in Chinese patients with BCS [11–14].

The germline constitutive JAK2 haplotype designated as GGCC or 46/1 haplotype is clearly associated with the acquisition of the JAK2 V617F mutation, and the JAK2 46/1 haplotype is a susceptibility factor for MPNs in Caucasian individuals [15–17]. Subsequently, a series of studies demonstrated that 46/1 haplotype was associated with the development of splanchnic vein thrombosis (SVT) [18–21]. Very recently, a meta-analysis of 26 observational studies involving 8,561 cases and 7,434 participants further indicated that JAK2 46/1 haplotype enrichment was significantly associated with the development of MPNs and SVT [22]. Of note, the majority of these studies were performed in Caucasian populations, only one study regarding the distribution of the JAK2 46/1 haplotype was completed in Chinese BCS patients [11]. Owing to the potential discrepancy of etiopathogenesis and treatment modalities in BCS patients [23, 24], further studies are needed in BCS patients of China.

Recently, Ohyashiki et al. [25] found that the minor C allele of JAK2 rs4495487, in addition to the JAK2 46/1 haplotype, contributed markedly to the occurrence of MPNs regardless of JAK2 genetic variations in the Japanese population. The contribution of rs4495487 was not reported in Caucasian population, but it is located between rs12343867 and 10974944. Thus, rs4495487 might be included in the 46/1 haplotype [25]. Taking into account ethnic discrepancies, we sought to demonstrate whether it was a risk factor for BCS patients in China.

The aim of this study, therefore, was to determine rs4495487 in relation to BCS risk and further study the association of single nucleotide polymorphism (SNP) with subtypes of BCS according to its location of obstruction and its prognosis role in high-prevalence region in China.

## 2. Materials and Methods

This retrospective study was conducted in the Affiliated Hospital of Xuzhou Medical College. From January 2010 to December 2014, a total of 300 BCS patients were consecutively recruited in this study. Patients who had secondary BCS were excluded. Meanwhile, 311 hospital-based subjects were randomly selected from Health Examination Center of the hospital as controls; none of them had a history of thrombosis, tumors, hypertension, liver disease, or diabetes mellitus. Information on demographic characteristics and clinical data was collected and confirmed through the medical records and self-administrated questionnaires. Individuals who smoked five or more cigarettes per day on average for >1 year were regarded as tobacco smokers, and subjects who consumed at least 3 alcoholic drinks per week for >1 year were considered to be drinkers. Child-Pugh score, model for end-stage liver disease (MELD) score, and BCS related prognostic indexes including Clichy score, Rotterdam score, and BCS-TIPS score were calculated as initially reported.

Patients were followed up until death, the end of this study period (December 2014), or the last visit data if the patient was lost to follow-up. Follow-up data were obtained from the medical archives, whenever possible, at prespecified intervals (1, 3, 6, and 12 months after discharging from hospital) or by telephone interview of the patients themselves or their relatives.

Approval was obtained from the ethics committee of the hospital for this study, and written informed consent was obtained in accordance with the Declaration of Helsinki.

### 2.1. Diagnosis and Definition

BCS was diagnosed using radiographic imaging (color Doppler ultrasonography, computed tomography, magnetic response imaging, and/or angiography) in accordance with previously published criteria [1]. According to the location of obstruction, BCS was classified into three groups: hepatic vein occlusion type, inferior vena cava occlusion type, and combined occlusion of hepatic vein and inferior vena cava [26]. BCS was considered secondary when the obstruction results from invasion or compression by tumor, abscesses, cysts, or parasitic mass [27].

### 2.2. Blood Sampling and JAK2 46/1 Genotyping Analysis

Genomic DNA was isolated from peripheral blood at admission using the UltraPure Genomic DNA Purification Kit (SBS, Shanghai, China). DNA samples were stored at −70°C until analysis. JAK2 V617F mutation was detected by allele-specific polymerase chain reaction (AS-PCR). DNA samples were genotyped by quantitative real-time polymerase chain reaction (qRT-PCR) on ABI 7900HT Fast RT-PCR System using a TaqMan SNP assay for rs4495487 polymorphism. The Assay ID for rs4495487 of the genotyping assays from Applied Biosystems was C_30016879_20, Applied Biosystems. For quality control, genotyping was performed by experiments blinded to the status of cases and controls, and a random selection of 10% samples was genotyped for repeat assays, with a reproducibility of 100%.

### 2.3. Statistical Analysis

All data were analyzed using SPSS version 16.0 software (Chicago, IL, USA) for windows. Normal testing was conducted by Kolmogorov-Smirnov test for quantitative variables. Normally distributed variables were summarized as mean ± standard deviation (SD); otherwise they were expressed with medians and interquartile range (25–75 percentiles). Comparisons between groups of quantitative variables were performed using Student's *t*-test when variable distributions were normal and using Mann-Whitney test in other cases. Categorical variables were summarized as percentage and were compared using chi-square test or the Fisher exact test, as appropriate. The Hardy-Weinberg equilibrium (HWE) was tested and examined with a chi-square test to compare the observed genotype frequencies to those expected for a population among controls. Furthermore, the strength of association between 46/1 haplotype and BCS was evaluated by calculating odds ratios (OR) and corresponding 95% confidence intervals (CI) using logistic regression, adjusted for age and gender. Survival curves were calculated by the Kaplan-Meier and comparison of survival functions among different genotypes was based on log-rank testing. The multivariate Cox-regression analysis was applied to evaluate the prognostic value of this SNP with adjustment for age, gender, smoking status, alcohol consumption, and BCS types. All *P* values were two-tailed, and the level of significance was set at *P* value 0.05.

## 3. Results

### 3.1. Characteristics of the Study Population

Among the 300 BCS cases and 311 controls, genomic DNA was obtained from all the subjects. The characteristics of cases and controls were summarized in Table 1. The two groups appeared to be adequately matched on age and gender distributions. As shown in Table 1, no significant difference was observed on drinking status and oral contraceptives use between the two groups, but smoking rate was higher in BCS cases than in controls (*P* = 0.035, adjusted for sex).

### 3.2. Association of JAK2 rs4495487 Polymorphism with BCS Susceptibility

Overall, JAK2 V617F mutation was found in 2.33% (7/300) of the patients. A total of 280 (93.33%) BCS cases and 310 (99.68%) controls were genotyped successfully. Among controls, the genotype distribution of rs4495487 was confirmed to be in HWE (*P* > 0.05). The frequencies of this polymorphism in cases and controls were presented in Table 2. Genotype frequencies of cases and controls were similar. Overall, in BCS patients, there was no statistical difference in frequency of the minor C allele compared with the controls (20.0% versus 18.7%; *P* = 0.57).

However, the stratification related to the presence of the JAK2 V617F mutation indicated that the minor C allele frequency of rs4495487 was significantly higher in individuals harboring JAK2 V617F mutation than in controls (42.9% versus 18.7%; *P* < 0.01). No difference in C allele frequency was found in JAK2 V617F negative BCS patients compared with controls (19.4% versus 18.7%; *P* = 0.57). Compared with TT genotype, the significantly elevated risk of JAK2 V617F positive patients with the CC genotype was 13.60 (95% CI: 2.04–90.79). In the recessive model, when CT/TT genotypes were used as the reference group, the CC genotype significantly increased JAK2 V617F positive BCS risk with OR of 12.00 (95% CI: 2.07–69.52).

When stratified by BCS types, we also observed a significantly increased risk of combined-type BCS associated with CC genotype in the recessive model (OR = 4.44, 95% CI = 1.31–15.12).

### 3.3. Clinical Characteristics Related to JAK2 rs4495487 Polymorphism in BCS Patients

We detected the association between JAK2 rs4495487 polymorphism and clinical characteristics (Table 3). Higher levels of platelet count (*P* = 0.007) were observed in BCS patients with CC genotype compared with individuals with the common TT genotype. In the dominant model, we found lower levels of carcinoembryonic antigen (*P* = 0.014). There were no differences among genotypes subgroups in the severity of liver disease at the time of diagnosis as determined by the Child-Pugh or MELD scores. Additionally, no difference was detected in the percentage of patients treated with angioplasty/stenting and transjugular intrahepatic portosystemic shunt among CC, CT, and TT genotypes (86.67%, 91.46%, and 91.80, resp.; *P* = 0.792). The others underwent alone medical therapy with anticoagulants and diuretics.

### 3.4. Association of JAK2 rs4495487 Polymorphism with the Survival of BCS Patients

**Figure 1:**
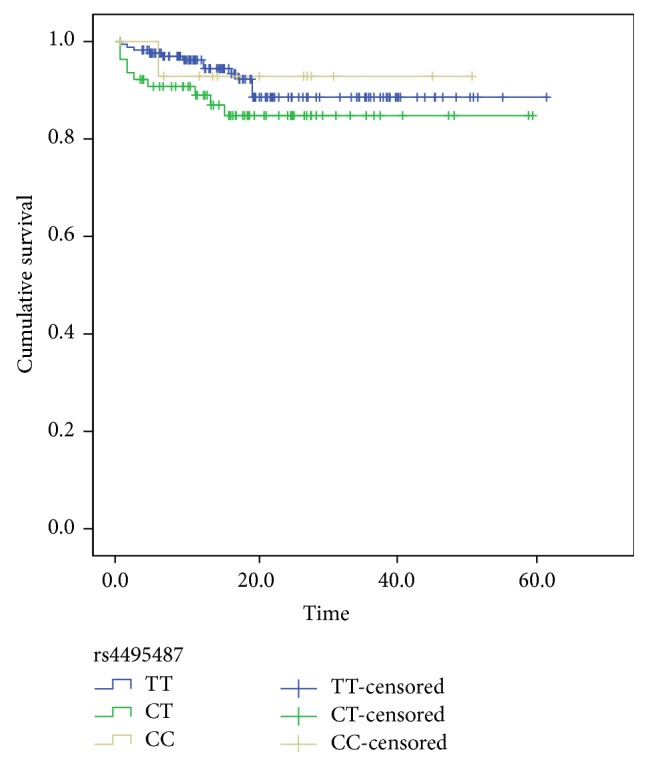
Kaplan-Meier estimates of overall survival by JAK2 rs4495487 genotypes (*P* = 0.299).

The median follow-up time was 17.8 months (range, 0.5 to 61.3). Ten patients were lost to follow-up after a median follow-up time of 7 months (range, 3 to 29). During follow-up, 28 patients died (hepatocellular carcinoma 7, hepatic encephalopathy 7, variceal bleed 6, gastrointestinal bleeding 3, and liver failure 5). Overall survival of BCS patients was analyzed using Kaplan-Meier survival curve for dependence on 46/1 genotypes. No significant difference was observed among different genotypes (Figure 1).

To adjust this curve for any other factors that may have affected on survival, we used the Cox proportional hazards model to analyze age, gender, tobacco smoking, alcohol consumption, BCS types, and 46/1 genotypes. In the multivariate analyses, only smoking status and BCS types demonstrated significant association with the outcome in BCS patients (Table 4).

## 4. Discussion

In the current study, we firstly identified the relationship between polymorphism of JAK2 rs4495487 and BCS in high-risk Chinese population. We found that JAK2 polymorphism was not associated with BCS susceptibility. Our observations were at variance with those reported by some researchers [19, 28], who found that 46/1 haplotype is present more frequently in BCS patients. However, the significant difference disappeared when including portal vein thrombosis (PVT) patients. This phenomenon could be explained because JAK2 V617F mutation prevalence in BCS patients was higher than that in PVT patients [7]. At the same time, the viewpoint from another point confirmed our result that JAK2 V617F mutation frequency was low in Chinese BCS patients. Some studies reported the JAK2 V617F mutation was found to be related to 46/1 haplotype enrichment, but the association and their relationship remain largely unknown. Two alternative explanations have been proposed: the “hypermutability hypothesis” that suggests genetic instability within the JAK2 gene [29] or the “fertile ground hypothesis” that suggests that carriers of 46/1 haplotype confer a selective advantage to JAK2 V617F positive clone and then manifest as a tendency for thrombosis [30]. The specific mechanisms need to be further researched.

To explore whether JAK2 V617F mutation was associated with JAK2 rs4495487 polymorphism in different subgroups in China, further analysis was performed according to JAK2 V617F status. In the subgroup of patients with JAK2 V617 mutation, we observed clear relationships between JAK2 rs4495487 polymorphism and the development of BCS. While we are aware that the group of JAK2 V617F mutation patients is small, this finding is in line with the study by Smalberg et al. [19], in which the 46/1 haplotype elevated the risk of occurrence of BCS patients with JAK2 V617F mutation compared with controls in an allele-dependent way. Why is the incidence of JAK2 rs4495487 CC genotype higher in BCS patients with JAK2 V617F mutation? The rs4495487 CC genotype may be not enough in itself to cause disease and contribute to BCS by the modulation of JAK2 gene expression or a specific modification of protein function through unidentified SNPs. However, given the low frequency of patients with JAK2 V617F mutation and the confidence intervals produced by the statistical analysis, the JAK2 rs4495487 polymorphism as a tool in the diagnostic work-up of Chinese BCS patients was challenged. Thus, the result should be interpreted with caution, and more studies on the prevalence JAK2 V617F mutation should be actively performed in China to confirm the finding. In addition, we noted that BCS patients with combined occlusion of hepatic vein and inferior vena cava were associated with the rs4495487 SNP in the recessive model. Result of this analysis may be due to the limited number of included patients, which requires further validity in larger and multicenter studies.

We observed that platelet count was higher in patients with homozygous carriers of JAK2 rs4495487 compared with individuals the TT genotype. While the previous study reported that hemoglobin, red cell count, and hematocrit were higher compared with 46/1 noncarriers, on the other hand, in one study, carriers of 46/1 grew significantly fewer granulocyte-macrophage colony forming units [17], in accordance with the concept that 46/1 haplotype might functionally differ from other JAK2 alleles. Although the platelet counts are all actually low and the higher the platelet count is then the less severe a disorder is suggested given that the thrombocytopenia is consumptive. The JAK2 V617F mutation can cause higher levels of both of leukocytes and erythrocytes by constitutively activating JAK2 kinase which regulates JAK-STAT signaling pathway, which changes the rheological properties of blood and stimulates platelet [31, 32]. Therefore, it is conceivable to suggest that JAK2 rs4495487 polymorphism can contribute to functional differences in JAK2 signaling related to a hypercoagulability state and development of BCS.

The polymorphism of JAK2 4495487 did not reveal a significant correlation with BCS patient survival in the study. The possible reason is that overall survival is determined not only by factors involving in BCS disease activity, but also by potential diseases and other complications. Thus, SNP alone may not be sufficient to affect the complex outcome.

In our study, some limitations of the present study need to be addressed. First, the low frequency of BCS patients with JAK2 V617F mutation severely restricts the reliability and usefulness of the analysis in the light of JAK2 rs4495487 polymorphism associated with BCS patients harboring mutation. However, all of patients were consecutively admitted to our hospital, which might decrease the potential bias of our result. Additionally, the Affiliated Hospital of Xuzhou Medical College is the largest hospital with 4,000 beds in Huaihai economic zone, China (http://www.jsxyfy.com/s/21/t/3/p/1/c/40/d/65/list.jspy). Owing to the extensive clinical experience in the interventional treatment for BCS, a larger number of patients from various regions of our country are admitted to the Department of Interventional Radiology. Second, the duration for follow-up of the patients was relatively short, with a median of 15.80 months, and warrants further research.

In summary, our findings exclude the hypothesis that the JAK2 rs4495487 confers susceptibility to BCS in a high-risk Chinese population. Rather, our observations indicate that the JAK2 rs4495487 is susceptibility factor JAK2 V617F positive BCS and combined-type BCS. These current findings should be verified in further larger studies with more rigorous designs of other Asian countries.

## Figures and Tables

**Table 1:** Baseline characteristics of enrolled cases and controls.

Variables	BCS cases [*n* (%)]	Controls [*n* (%)]	*t* or *χ* ^2^	*P* value
Overall	300	311		
Age				
Mean ± SD (year)	44.52 ± 13.40	43.17 ± 11.05	−1.570	0.116
Gender				
Male	163 (54.33)	165 (53.05)	0.100	0.751
Female	137 (45.67)	146 (46.95)		
Tobacco smoking				
Yes	88 (29.33)	65 (20.90)	5.785	0.016^*∗*^
No	212 (70.67)	246 (79.10)		
Alcohol consumption				
Yes	70 (23.33)	86 (27.65)	1.498	0.221^†^
No	230 (76.67)	225 (72.35)		
Oral contraceptives				
Yes	13 (9.49)	16 (10.96)	0.166	0.684
No	124 (90.51)	130 (89.04)		

BCS: Budd-Chiari syndrome; SD: standard deviation.

^*∗*^ *P* = 0.035 adjusted for sex. ^†^ *P* = 0.383 adjusted for sex.

**Table 2:** Association between JAK2 rs4495487 polymorphism and Budd-Chiari syndrome patients.

	*N*	rs4495487 genotype			C allele	*P*	Odds ratio (95% CI)		
		CC	CT	TT	frequency		CC versus TT	CC/CT versus TT	CC versus CT/TT
Controls	310	10	96	204	0.187				
Overall	280	15	82	183	0.200	0.575	1.67 (0.73–3.81)	1.02 (0.73–1.43)	1.70 (0.75–3.84)
JAK2 status									
JAK2-positive	7	3	2	2	0.429	<0.01	13.60 (2.04–90.79)	2.57 (0.56–11.68)	12.00 (2.07–69.52)
JAK2-negative	273	13	80	180	0.194	0.760	1.47 (0.63–3.44)	0.99 (0.71–1.40)	1.50 (0.65–3.48)
Type of BCS									
IVC	173	9	55	109	0.211	0.370	1.61 (0.64–4.08)	0.98 (0.66–1.45)	1.65 (0.66–4.13)
HV	68	2	24	42	0.206	0.613	0.97 (0.21–4.60)	1.17 (0.68–2.02)	0.91 (0.20–4.25)
Com	39	4	7	28	0.192	0.912	3.02 (0.89–10.31)	0.84 (0.41–1.73)	4.44 (1.31–15.12)

BCS: Budd-Chiari syndrome; HV: hepatic vein; IVC: inferior vena cava; Com: combined obstruction of hepatic vein and inferior vena cava.

*P*: *P* value for C-allele frequency comparisons.

**Table 3:** Laboratory characteristics at presentation for Budd-Chiari syndrome patients with the JAK2 rs4495487 polymorphism.

	rs4495487 genotype			*P*1 value	*P*2 value	*P*3 value
	CC	CT	TT			
Age (years)	43.53 (30.39–56.67)	44.33 (30.76–57.90)	44.71 (31.48–57.94)	0.934	0.743	0.763
Male (%)	9 (60.00)	38 (46.34)	106 (57.92)	0.197	0.875	0.130
Alanine aminotransferase (U/L)	22.0 (18.0–33.0)	21.0 (16.0–32.0)	21.0 (16.0–29.0)	0.950	0.810	0.940
Aspartate aminotransferase (U/L)	28.0 (22.0–39.0)	29.5 (22.0–41.0)	30.0 (24.0–43.0)	0.725	0.620	0.423
Platelets (10^9^/L)	137.0 (105.0–221.0)	108.0 (75.0–160.0)	93.0 (70.0–133.0)	**0.019**	**0.007**	0.530
White blood cell (10^9^/L)	3.43 (2.89–3.88)	3.58 (2.78–4.98)	3.94 (2.97–5.38)	0.538	0.286	0.292
Red blood cell (10^9^/L)	4.63 (3.68–5.63)	4.18 (3.68–4.65)	4.17 (7.72–4.64)	0.699	0.324	0.865
Hemoglobin (g/L)	120.0 (111.0–135.5)	118.5 (102.0–141.0)	120.0 (103.0–134.0)	0.762	0.560	0.478
Carcinoembryonic antigen (ng/mL)	1.74 (0.95–2.97)	1.78 (0.92–2.42)	2.11 (1.32–3.45)	**0.049**	0.322	**0.014**
Child-Pugh score^*∗*^	7 (6–13)	6 (5–12)	7 (6–12)	0.171	0.410	0.295
MELD score	9.26 (5.20–12.63)	7.94 (5.08–11.72)	8.80 (5.44–12.32)	0.584	0.890	0.393
Clichy score	5.48 (4.52–6.35)	4.93 (4.31–6.16)	5.24 (4.52–6.26)	0.218	0.910	0.136
Rotterdam score	1.12 (0.19–1.19)	1.11 (0.12–1.19)	1.14 (0.23–1.22)	0.154	0.886	0.079
BCS-TIPS score	4.53 (3.90–5.27)	4.31 (3.74–5.13)	4.40 (3.68–5.17)	0.699	0.513	0.882

^*∗*^Data are medians, with ranges in parentheses.

Continuous data are expressed as median (25–75 percentiles) and categorical data as frequencies (percentage).

*P*1: *P* value genotype comparisons (CC/CT/TT); *P*2: *P* for CC versus TT; *P*3: *P* value for CC/CT versus TT genotype comparisons.

MELD: model for end-stage liver disease; TIPS: transjugular intrahepatic portosystemic shunt.

**Table 4:** Cox multivariate regression analysis of potential factors for overall survival in BCS patients.

Variables	HR (95% CI)	*P* value
Age	0.990 (0.956–1.026)	0.576
Gender		
Male	1	
Female	1.208 (0.385–3.791)	0.746
Tobacco smoking		
No	1	
Yes	1.391 (1.025–1.884)	0.030
Alcohol consumption		
No	1	
Yes	0.457 (0.114–1.829)	0.268
rs4495487 polymorphism		
TT	1	
CT	2.021 (0.794–5.114)	0.140
CC	1.673 (0.212–13.183)	0.625
Types of BCS		
IVC	1	
HV	3.557 (1.164–10.875)	0.026
Com	4.246 (1.188–15.167)	0.026

BCS: Budd-Chiari syndrome; HV: hepatic vein; IVC: inferior vena cava; Com: combined obstruction of hepatic vein and inferior vena cava; HR: hazard ratio.
